# Is Ethics Approval Necessary for all Trials? A Clear But Not Certain Process

**DOI:** 10.4274/Mirt.80664

**Published:** 2013-12-10

**Authors:** F. Suna Kıraç

**Affiliations:** 1 Pamukkale University Medical School, Department of Nuclear Medicine, Denizli Turkey

**Keywords:** Ethics approval, ethics guidelines, ethical directives

All researchers want to do well planned study and their trial to be published and globally read. However, most of researchers feel there is generally a lack of guidance on how to prepare good papers and what the rules of ethics at the beginning of study. Thus, they seek to learn medical publication rules and find out the advices of the Helsinki Declaration which is prepared in line with WHO policies and also guidelines on publication ethics (international and /or national) (1,2,3,4,5,6,7). They define that good planning, design, management of the study and safely recording of all study data are main principles for good publication. Looking at good publication principles in detail, ethics approval and informed consent seem to be as important beginning procedures for a study. The Helsinki Declaration, lastly published on Oct 2008, declares that ethical approval for all interventional studies is required (1,2) and the Committee on Publication Ethics (COPE ) provides valuable advices to researchers on publication ethics (3). Here, ethics approval will be mainly discussed rather than informed consent because of some confusions arising due to ethics directives recently published in our country (4,5). 

The requirement for ethics approval during submission to publish is depended on the local and international publication guidelines, some international and national journals ask it for all submitting papers, even retrospective studies, but some of them not. Many journals generally present to authors whether ethics approval is necessary or not. However, sometimes clear explanation cannot be achieved and authors raised the question which frequently occurs for the retrospective studies and case reports. In this paper, we wish to discuss this controversial issue and to share last codes published by government in our country and international guidelines such as Consolidated Standards of Reporting Trials (CONSORT) and Uniform Requirements for Manuscripts Submitted to Biomedical Journals (URM) which state the ethical principles in the conduct and reporting of research and provide recommendations relating to specific parts of publishing (6,7).

According to international guides, ethics approval is legally required when human and animals are to be used for teaching or new intervention research, however, it is not receiving informed consent from all participitants accompanied with ethics approval is necessary for a prospective randomised trial. Last version of the Declaration of Helsinki published at Oct. 2008 has described that physicians should consider the ethical, legal and regulatory norms and standards for research involving human subjects in their own countries as well as applicable international norms and standards. And also, it says that the research protocol must be submitted for consideration, comment, guidance and approval to a research ethics committee before the study begins. This committee must take into consideration the laws and regulations of the country or countries in which the research is to be performed as well as applicable international norms and standards but these must not be allowed to reduce or eliminate any of the protections for research subjects set forth in this Declaration (1). The lack of consent and approval for the research study suggests a breach of the Helsinki declaration.

In Turkey, founding of the national and local ethics committees and regulation of the rules for publication ethics became actual with respect to the National Code of Clinical Research which is recently published on 13th April, 2013 (5). This ordinance describes how local ethical committee are does work, and what its” responsibility and competency as well as study types and main requirements for research planning essentially based on the international rules. Research ethics committees (individually for invasive clinical research, non-invasive clinical trials and animal research) are established in proper institutions and publish their own ethics instructions in accordance with applicable codes and guidelines. These institutional directives are basically prepared with respect to Code of Clinical Research published on 19 July, 2011 and declare that all invasive clinical trials and animal studies must be submitted for comment, approval and guidance to a relevant ethics committee before the study begins. As for last published national ethics code, ethics approval is obligatory for all prospective interventional studies and they should have been consulted with ethics committee (local or governmental) before starting. However, retrospective studies (clinical or not) are obviously excluded from this code (5) with the major difference from the previous one published in 2011 (4). Although recently published Code of Clinical Research declared that retrospective archive studies do not need ethics approval, all institutional ethics committee instructions still ask it for submission of scientific publication in our country (5). At this time, the method of prospective interventional studies is clear, but an important struggle for noninvasive retrospective clinical studies has developed. This discrepancy between national and local ethics directives may result in a serious confusion during publication process, and, an important question arises in the mind of researchers and peer-reviewers. To solve this problem, it is suggested that the local instructions should be revised according to lastly published national code on ethics for clinical trials as early as possible. Until publishing revised versions of local instructions, if doubt exists whether the retrospective study was conducted in accordance with the Helsinki Declaration because of lacking of ethics approval, it may be necessary that the authors having to explain the rationale basis for their approach according to The National Code of Clinical Trials.

Another important issue for good medical publication is informed consent process. The ethical meaning of informed consent is the principle of respect for persons. Receiving informed consent is based on the international guidelines and national standards, like ethics approval. Informed consent must be read and signed by the patient who accepts to enter the study, by a witness who is a relative or friend of the patient, and also by a staff member having a part in the study. However, depending on National Code on the Patient Rights (published on Aug 01,1998), all patients must sign informed consent forms not only for specific trials but also for each medical application (diagnostic or therapeutic) in our country (9). When the patient is not legally responsible, like children and unconscious patients who are physically or mentally incapable of giving consent, the surrogate decision-maker is responsible for signing this form on the patient’s behalf. Though informed consent for research is important, the fact that a participant or surrogate may be willing to give consent to a research does not, in itself, mean that the research is ethically acceptable (1,8). Informed consent should be accompanied with ethical approval in interventional studies. Declaration of Helsinki notifies that medical study using human material or data, physicians must normally seek consent for the collection, analysis, storage and/or reuse. If getting of consent would be impossible or impractical for research or would pose a threat to the validity of the research, the study may be done only after submitting to consideration and approval of a proper ethics committee (1).

In conclusion, instutional ethics committee approval and informed consent are obligatory parts in all interventional studies (human or animal) without any doubt. However, recently published The National Code on Clinical Trials has declared that ethics approval is not necessary for real retrospective studies. After publishing this last version of ethics code, retrospective studies and cases which do not have ethics committee approval are acceptable to be reviewed in the most of medical journals though rarely it may not be the case. On the other hand, publication ethics rules are likely to be changed by ethics committees and ethics approval may be asked for all studies in the future, like informed consent. To avoid stumbling block during publication process later, all trials even retrospective ones are advised to be submitted to a proper ethical committee before beginning.
